# Supplementary material for: Global evidence of positive biodiversity effects on spatial ecosystem stability in natural grasslands
Source: Nat Commun. 2019 Jul 19;10:3207. doi: 10.1038/s41467-019-11191-z (PMC6642091; doi:10.1038/s41467-019-11191-z)
Supplement: Supplementary file 1 — Supplementary Information [file 41467_2019_11191_MOESM1_ESM.pdf]

Supplementary Information for

Global evidence of positive biodiversity effects on spatial ecosystem stability in natural grasslands

Wang *et al*.

This file includes:

Supplementary Figure 1 to Figure 6

Supplementary Table 1 to Table 2

Supplementary Note 1 to Note 2

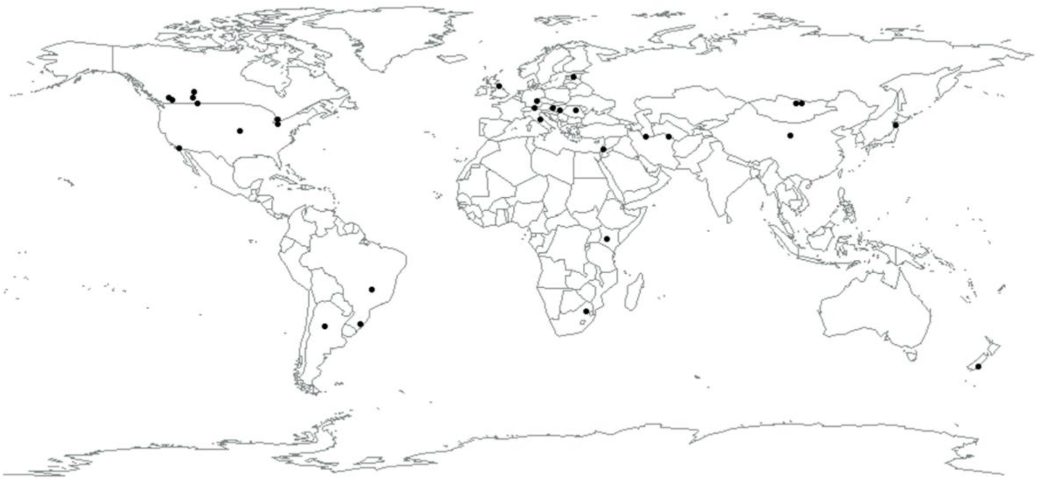

Supplementary Figure 1 | Locations of the 30 HerbDivNet sites that provided data for this study. Each black point represents the geographic centroid of each study site, which includes 2 to 14 sampling grids. Source data are provided as a Source Data file.

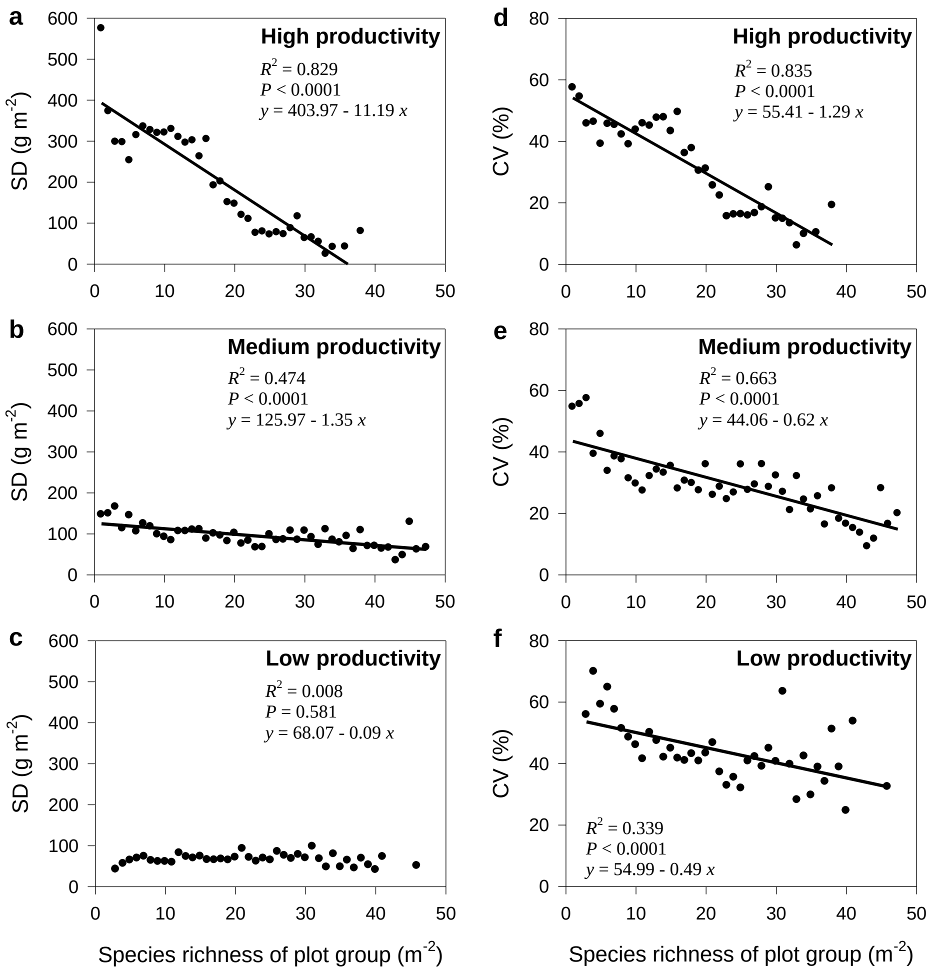

Supplementary Figure 2 | Simple regression models for the global relationship between biodiversity and spatial variability along productivity gradient. Spatial variability in grassland productivity was measured as standard deviation (*SD*, **a-c**) and coefficient of variation (*CV*, **d-f**) across plots with the same species richness for three different levels of productivity separately. The division of productivity gradients is the same as that of Figure 3, where the 151 grids were divided into three equal groups depending on their grid-level mean productivity: low, medium, and high productivity, with 50 to 51 grids each. The black line represents the fitted relationship. For each productivity level, species richness was used to group plots containing one, two, three species, etc. If a group has less than three plots, it was combined with the next group to ensure there were three or more plots in each group. Source data are provided as a Source Data file.

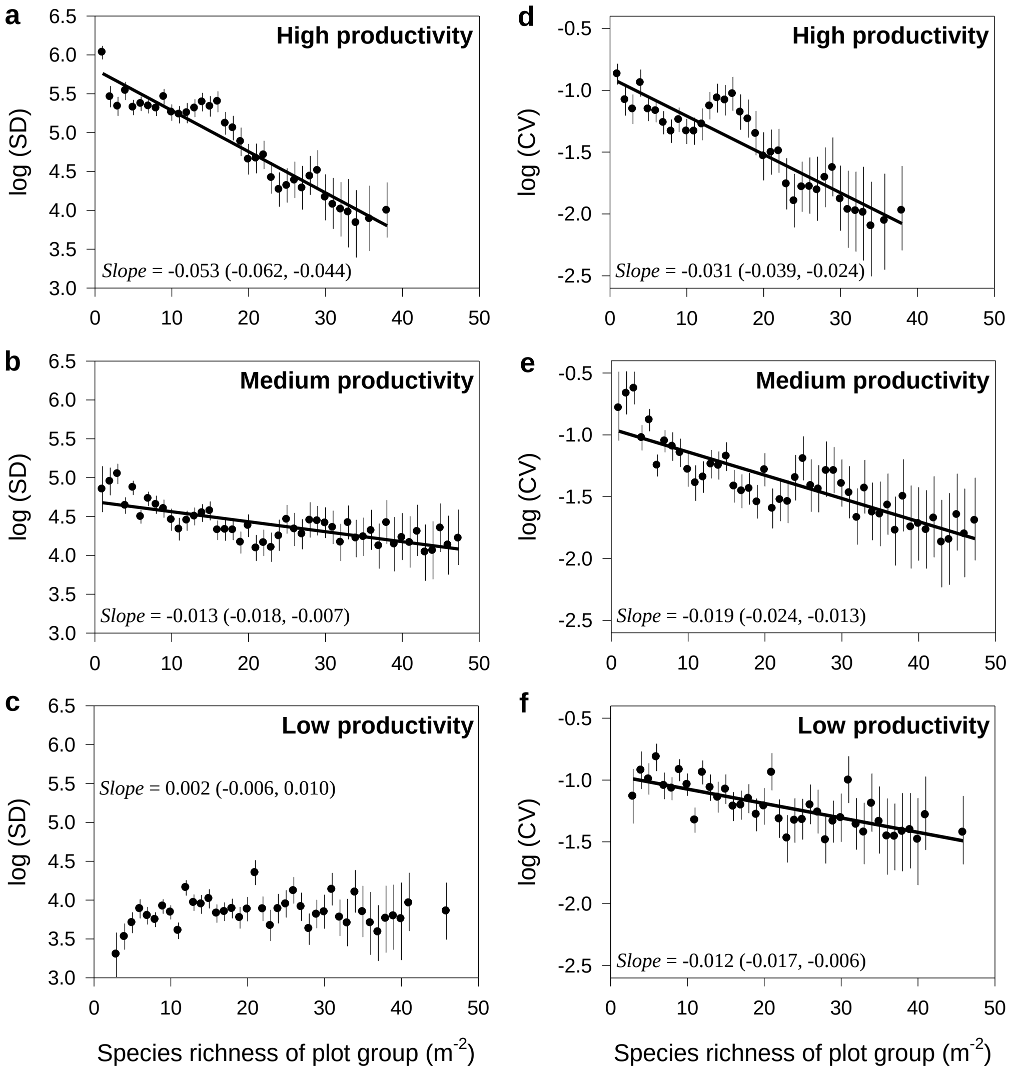

Supplementary Figure 3 | Hierarchical Bayesian models for the global relationship between biodiversity and spatial variability along productivity gradient. Black points and vertical lines show the means and 95% credible intervals (CI) of log-transformed productivity variation for each richness level. Spatial variability in grassland productivity was measured as standard deviation (*SD*, **a-c**) or coefficient of variation (*CV*, **d-f**) across plots with the same richness for three different levels of productivity (low, medium, and high), respectively. The division of productivity gradients is the same as that of Figure 3. For each productivity level, species richness is used to group plots as in Supplementary Figure 2. The black line represents the fitted relationship. The text shows the median and 95% CI of the slope. Source data are provided as a Source Data file.

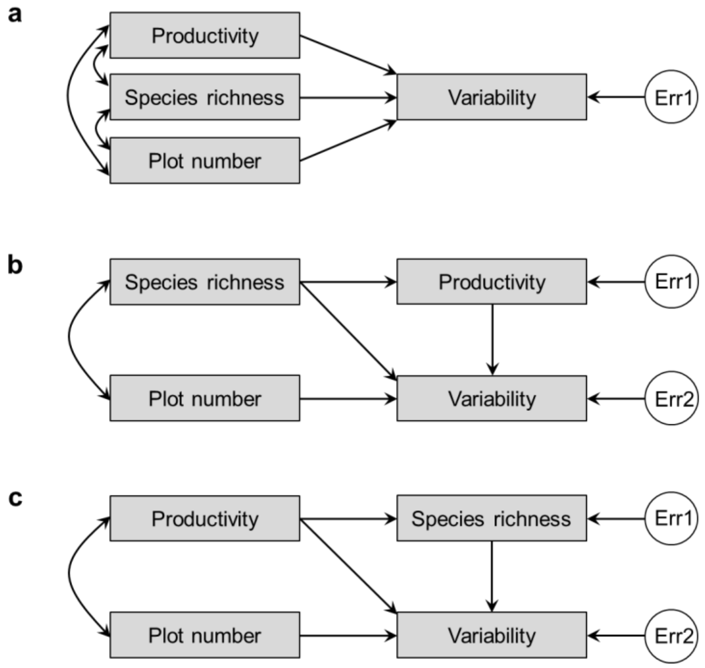

Supplementary Figure 4 | Three competing structural models. (**a**) Mean productivity, species richness and plot number were independent variables, productivity variability (measured as either *SD* or *CV*) was a response variable. (**b**) Species richness and plot number were independent variables, productivity and variability were intermediate and response variables, respectively. (**c**) Productivity and plot number were independent variables, species richness and variability were intermediate and response variables, respectively. The exogenous unobserved variables err1 and err2 account for the unexplained error in the estimation of the endogenous variables in the models.

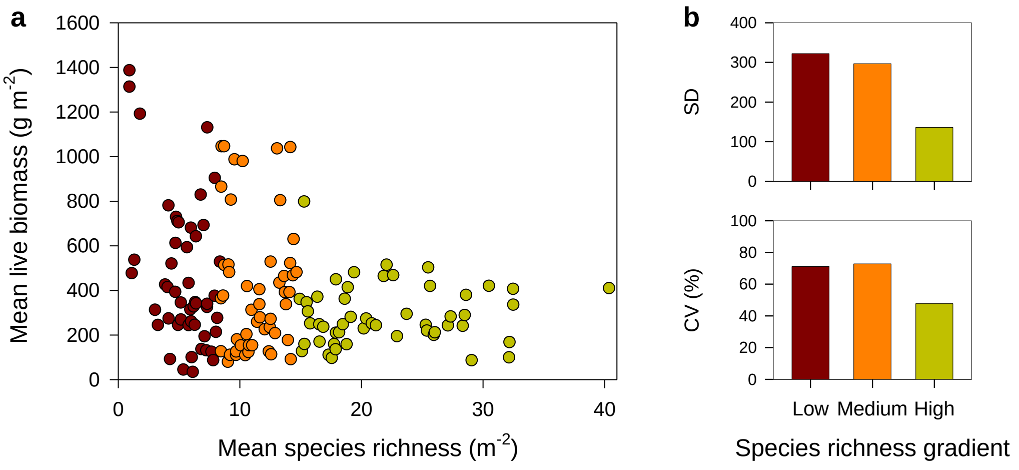

Supplementary Figure 5 | Estimating the effects of species richness on productivity variability at the grid-level. (**a**) Relationship between mean species richness and mean productivity at each grid for HerbDivNet data ($N = 151$ grids). 151 grids were divided into three equal-number diversity classes (low, medium, and high, with 50 to 51 grids each), corresponding to different colors. (**b**) Histograms comparing the variability in grid-level mean productivity for three diversity classes. Variability was measured as either standard deviation (*SD*) or coefficient of variation (*CV*) of mean productivity for each diversity class, respectively. Each bar corresponds to the points (grids) of the same color in **a**. Source data are provided as a Source Data file.

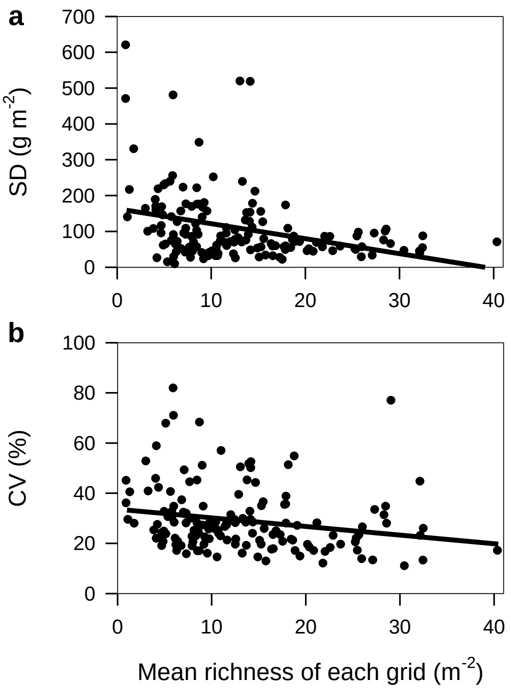

Supplementary Figure 6 | Within-grid variability in productivity that respond to the mean species richness. Scatterplots showing the linear relationships between plot productivity variability within each grid and the grid-level average species richness for HerbDivNet data ($N = 151$ grids). (**a**) Variability was measured as standard deviation (SD) of productivity across plots within grids. Linear regression equation is reported for ordinary least-squares model: $SD = 163.30 - 4.18 \times richness$, $R^2 = 0.110$, $P < 0.0001$; (**b**) Variability was measured as coefficient of variation (CV) of productivity across plots within grids. Linear regression equation is reported for ordinary least-squares model: $CV = 33.62 - 0.34 \times richness$, $R^2 = 0.043$, $P < 0.0001$. Source data are provided as a Source Data file.

Supplementary Table 1 | Summary statistics for parameters in the hierarchical Bayesian model using standard deviation as a measure of productivity variability

Parameter	Median	2.5% quantile	97.5% quantile
β_0	5.197	5.045	5.346
β_1	-0.031	-0.036	-0.024
$\gamma_{1,0}$	73.060	43.131	101.996
$\gamma_{1,1}$	-0.272	-0.338	-0.205
σ_{α_0}	229.472	204.418	261.290
σ_{α_1}	82.499	69.875	97.411
σ_β	0.239	0.185	0.311

β_1 represents the effect of richness on productivity variation. $\gamma_{1,1}$ assessed how the richness effects on productivity vary across stress or productivity gradients. The meanings of other parameter symbols can be found from equations 1-4 in the Methods.

Supplementary Table 2 | Fitted measures for the competing structural equation models a–c listed in Supplementary Figure 4

Fitting index	Evaluation criterion	Models		
		a	*b*	*c*
Absolute fit indices				
χ^2	$P > 0.05$	$P = 0.005$	$\boldsymbol{P = 0.353}$	$P < 0.001$
GFI	> 0.90	**0.93**	**0.99**	0.74
AGFI	> 0.90	0.28	**0.91**	−1.58
RMSEA	< 0.08	0.38	**0.00**	1.08
Relative fit indices				
NFI	> 0.90	**0.95～0.96**	**0.99**	0.63～0.70
RFI	> 0.90	0.68～0.75	**0.97**	−1.23～−0.77
IFI	> 0.90	**0.95～0.96**	**1.00**	0.63～0.71
TLI	> 0.90	0.71～0.77	**1.01**	−1.28～−0.80
CFI	> 0.90	**0.95～0.96**	**1.00**	0.62～0.70
Parsimonious fit indices				
NC	$1 < NC < 3$	7.79	0.86	54.69
AIC	The smaller the better	25.79	**18.86**	72.69
CAIC	The smaller the better	51.44	**44.52**	98.34

Number or numerical range in a box represents the combined results obtained when variability was measured as either *SD* or *CV*. Bold number indicates meeting the evaluation criterion (column 2). Model b is the best fitting model for HerbDivNet live biomass data. *GFI*: Goodness-of-fit index; *AGFI*: Adjusted goodness-of-fit index; *RMSEA*: Root mean square error of approximation; *NFI*: Normed fit index; *RFI*: Relative fit index; *IFI*: Incremental fit index; *TLI*: Tacker-Lewis index; *CFI*: Comparative fit index; *NC*: Normed chi-square (the minimum discrepancy divided by its degrees of freedom); *AIC*: Akaike information criterion; *CAIC*: Consistent Akaike information criterion.

Supplementary Note 1 | R code for Bayesian Models

```
############################################################
#
# JAGS code for running the hierarchical Bayesian model,
# which tests the (1) relationship between diversity and average
# biomass, (2) how diversity effect on biomass vary across
# productivity gradient and (3) the relationship between diversity
# and biomass variation.
# The code was tested with rjags 4-6 (linked to JAGS 4.3.0)
# in R 3.3.3.
# The code corresponds to the equation 1a-d in the main text.
# Programmer: Yuxin Chen (chenyux9@gmail.com)
# Version: 30th Jan., 2019
#
############################################################

#----------------------------------------------------------
# Variable note
# N_plot: number of plots
# N_richness: number of unique richness levels
# richness: species richness for a plot
# unique_richness: species richness for a unique richness level
# richness_position: index mapping a plot to its corresponding unique richness level
# uniq_rich_mu_biomass: mean biomass at each unique richness levels
# N_grid: number of grids
# grid: index for grid
# grid_biomass: grid-level average biomass
# N_site: number of sites
# site: index for site
# light: daylight hours
# temp: temperature
# precip: precipitation
# pp: Bayesian p value corresponding to plot-level difference between observed and predictive
biomass
# p_mean_biomass: Bayesian p value corresponding to difference in average biomass across plots
# p_cv_biomass: Bayesian p value corresponding to difference in CV of biomass across plots
#
#----------------------------------------------------------

model
{
# likelihood
for (i in 1:N_plot){
```

```
    biomass_mu[i] <- alpha_0[grid[i]] + alpha_1[grid[i]] * richness[i] + site[i]
    observ_biomass[i] ~ dnorm(biomass_mu[i], tau[richness_position[i]])
    simul_biomass[i] ~ dnorm(biomass_mu[i], tau[richness_position[i]])
    p_biomass[i] <- step(observ_biomass[i] - simul_biomass[i])
}

pp <- sum(p_biomass[]) / N_plot

# posterior predictive check
mean_biomass <- mean(observ_biomass[])
mean_biomass_simul <- mean(simul_biomass[])
cv_biomass <- sd(observ_biomass[]) / mean_biomass
cv_biomass_simul <- sd(simul_biomass[]) / mean_biomass_simul
p_mean_biomass <- step(mean_biomass - mean_biomass_simul)
p_cv_biomass <- step(cv_biomass - cv_biomass_simul)

for (j in 1:N_grid){
    alpha_0_mu[j] <- gamma0_0 + gamma0_1 * light[j] + gamma0_2 * temp[j] + gamma0_3 *
precip[j]
    alpha_0[j] ~ dnorm(alpha_0_mu[j], alpha_0_tau)
    alpha_1_mu[j] <- gamma1_0 + gamma1_1 * grid_biomass[j]
    alpha_1[j] ~ dnorm(alpha_1_mu[j], alpha_1_tau)
}

for (k in 1:N_richness){
    tau[k] <- pow(sd[k], -2)
    sd[k] <- exp(log_sd[k])
    log_sd[k] ~ dnorm(log_sd_mu[k], tau_tau)
    log_sd_mu[k] <- beta_0 + beta_1 * unique_richness[k]
}

for (l in 1:N_site){
    site[l] ~ dnorm(0, site_tau)
}

# prior
gamma0_0 ~ dnorm(0, 1.0E-6)
gamma0_1 ~ dnorm(0, 1.0E-6)
gamma0_2 ~ dnorm(0, 1.0E-6)
gamma0_3 ~ dnorm(0, 1.0E-6)
gamma1_0 ~ dnorm(0, 1.0E-6)
gamma1_1 ~ dnorm(0, 1.0E-6)
alpha_0_tau ~ dgamma(0.001, 0.001)
alpha_1_tau ~ dgamma(0.001, 0.001)
```

```
beta_0 ~ dnorm(0, 1.0E-6)
beta_1 ~ dnorm(0, 1.0E-6)
tau_tau ~ dgamma(0.001, 0.001)

site_tau ~ dgamma(0.001, 0.001)

alpha_0_sd <- pow(alpha_0_tau, -0.5)
alpha_1_sd <- pow(alpha_1_tau, -0.5)
sd_sd <- pow(tau_tau, -0.5)
site_sd <- pow(site_tau, -0.5)
}
```

Supplementary Note 2 | R code for Structural Equation Models

```
###############################################################################
# Programmer: Nayun Shi (shiny@mail2.sysu.edu.cn)
# Version: 5 November 2018

# Load required libraries
library(lavaan) # Version 0.6-3

####### Structural Equation Models for biodiversity and productivity #######
# Read in data
semdata <- read.csv("semdata1.csv")

# List structured equations for lavaan
semmodel <- '
   # regression
   biodiversity ~ light_availability + moisture + temperature
   productivity ~ biodiversity + light_availability + moisture + temperature
   # correlation
   light_availability ~~ moisture
   light_availability ~~ temperature
   moisture ~~ temperature
'

# Fit SEM model
sem.test <- sem(semmodel, data = semdata) ## general
sem.test1 <- sem(semmodel, data = semdata, group="class") ## multigroup

# Summary output
summary(sem.test, fit.measures = T)
standardizedsolution(sem.test)
inspect(sem.test, "r2")
#same with multigroup model
summary(sem.test1, fit.measures = T)
standardizedsolution(sem.test1)
inspect(sem.test1, "r2")

# The code corresponds to the Figure 2 and Figure 3 in the supplementary text.


###### Structural Equation Models for species richness, live biomass and biomass variability
######
# Read in data
semdata <- read.csv("semdata2.csv")
```

```
# List structured equations (SD)
SDmodel <- '
   SD ~ live_biomass + species_richness + plot_number
   live_biomass ~ species_richness + plot_number
   species_richness ~~ plot_number
'
# Fit SEM model
SDmodel.test <- sem(SDmodel, data = semdata2)

# Summary output
summary(SDmodel.test, fit.measures= T)
standardizedsolution(SDmodel.test)
inspect(SDmodel.test, "r2")

# List structured equations (CV)
CVmodel <- '
   CV ~ live_biomass + species_richness + plot_number
   live_biomass ~ species_richness + plot_number
   species_richness ~~ plot_number
'
# Fit SEM model
CVmodel.test <- sem(CVmodel, data = semdata2)

# Summary output
summary(CVmodel.test, fit.measures= T)
standardizedsolution(CVmodel.test)
inspect(CVmodel.test, "r2")

# The code corresponds to the Figure 6 in the supplementary text.
```
