# Supplementary material for: Global evidence of positive biodiversity effects on spatial ecosystem stability in natural grasslands
Source: Nat Commun. 2019 Jul 19;10:3207. doi: 10.1038/s41467-019-11191-z (PMC6642091; doi:10.1038/s41467-019-11191-z)
Supplement: Supplementary file 3 — Reporting Summary [file 41467_2019_11191_MOESM3_ESM.pdf]

nature research

Corresponding author(s): Yongfan Wang; Michel Loreau

Reporting Summary

Nature Research wishes to improve the reproducibility of the work that we publish. This form provides structure for consistency and transparency in reporting. For further information on Nature Research policies, see Authors & Referees and the Editorial Policy Checklist.

Statistical parameters

When statistical analyses are reported, confirm that the following items are present in the relevant location (e.g. figure legend, table legend, main text, or Methods section).

n/a	Confirmed	
☐	☒	The exact sample size (n) for each experimental group/condition, given as a discrete number and unit of measurement
☐	☒	An indication of whether measurements were taken from distinct samples or whether the same sample was measured repeatedly
☐	☒	The statistical test(s) used AND whether they are one- or two-sided<br>*Only common tests should be described solely by name; describe more complex techniques in the Methods section.*
☐	☒	A description of all covariates tested
☐	☒	A description of any assumptions or corrections, such as tests of normality and adjustment for multiple comparisons
☐	☒	A full description of the statistics including central tendency (e.g. means) or other basic estimates (e.g. regression coefficient) AND variation (e.g. standard deviation) or associated estimates of uncertainty (e.g. confidence intervals)
☐	☒	For null hypothesis testing, the test statistic (e.g. F, t, r) with confidence intervals, effect sizes, degrees of freedom and P value noted<br>*Give P values as exact values whenever suitable.*
☐	☒	For Bayesian analysis, information on the choice of priors and Markov chain Monte Carlo settings
☐	☒	For hierarchical and complex designs, identification of the appropriate level for tests and full reporting of outcomes
☐	☒	Estimates of effect sizes (e.g. Cohen's d, Pearson's r), indicating how they were calculated
☐	☒	Clearly defined error bars<br>*State explicitly what error bars represent (e.g. SD, SE, CI)*

Our web collection on statistics for biologists may be useful.

Software and code

Policy information about availability of computer code

Data collection	No software was used
Data analysis	SigmaPlot 10.0; R version 3.3.2

For manuscripts utilizing custom algorithms or software that are central to the research but not yet described in published literature, software must be made available to editors/reviewers upon request. We strongly encourage code deposition in a community repository (e.g. GitHub). See the Nature Research guidelines for submitting code & software for further information.

Data

Policy information about availability of data

All manuscripts must include a data availability statement. This statement should provide the following information, where applicable:
- Accession codes, unique identifiers, or web links for publicly available datasets
- A list of figures that have associated raw data
- A description of any restrictions on data availability

The grassland data is from Fraser et al. (2015), the data has been deposited by Fraser et al. in the Dryad repository (https://datadryad.org/resource/doi:10.5061/dryad.038q8; Title: raw plot data from globally distributed sites). Temperature and precipitation data are from WorldClim (http://worldclim.org/version2), and

daylight hours data is from 'NASA Surface Meteorology and Solar Energy: Global Data Sets' (https://eosweb.larc.nasa.gov/cgi-bin/sse/global.cgi). The source data underlying Figs 2, 3, 4a, b, 5, 6a–d, 7a, b and Supplementary Figs 1, 2a–f, 3a–f, 5a, b, 6a and b are provided as a Source Data file.

Field-specific reporting

Please select the best fit for your research. If you are not sure, read the appropriate sections before making your selection.

☐ Life sciences ☐ Behavioural & social sciences ☒ Ecological, evolutionary & environmental sciences

For a reference copy of the document with all sections, see nature.com/authors/policies/ReportingSummary-flat.pdf

Ecological, evolutionary & environmental sciences study design

All studies must disclose on these points even when the disclosure is negative.

Study description	To minimize the methodological differences that exist among disparate studies, a global survey of natural grasslands uses consistent data-collection methods for testing global hypotheses in ecology and environmental science, the Herbaceous Diversity Network (HerbDivNet) conducts coordinated surveys in 30 natural grasslands (sites) from 19 countries and six continents.
Research sample	HerbDivNet has 9,640 plots (each 1 m×1 m) distributed across 151 grids (each 8 m×8 m) in 30 natural grasslands (sites) from 19 countries and six continents. Grassland type is separated into 5 categories: temperate, temperate wet meadow, Mediterranean, tropical and subtropical, and alpine.
Sampling strategy	Although there is no sample-size calculation was performed, all HerbDivNet sites are located in areas dominated by herbaceous vegetation representing the regional species composition, and the sampling includes the main types of natural grassland on land.
Data collection	HerbDivNet is a network of researchers working at herbaceous grassland sites performing coordinated distributed observations. That is, ecologists who are willing to participate, selected suitable natural grasslands in their respective countries, collected species richness and biomass data according to a consistent sampling protocol. So far, HerbDivNet includes more than 66 participants from six continents and 19 countries.
Timing and spatial scale	Timing scale: 2013-present; Spatial scale: at the global extent.
Data exclusions	HerbDivNet has 10,048 plots. We used data from the 9,640 plots in which both species richness and aboveground live biomass were measured in our analysis.
Reproducibility	Although our work is not an experiment but a field survey, we have gained similar results from two scales (plot- and grid-level), that is, the role of species diversity is to stabilize productivity.
Randomization	This was not relevant to our study, because our work is not an experiment but field surveys.
Blinding	Blinding was not relevant to our study because our data came from field surveys.

Did the study involve field work? ☒ Yes ☐ No

Field work, collection and transport

Field conditions	Our field survey sites are located in 19 countries on six continents around the world, and the environmental conditions vary greatly from site to site.
Location	Our field survey sites are located in 19 countries on six continents around the world.
Access and import/export	We obtained the HerbDivNet data from publically available sources. In addition, one of our co-authors, L.H.Fraser, is the HerbDivNet PI.
Disturbance	None

Reporting for specific materials, systems and methods

Materials & experimental systems

n/a	Involved in the study
☒	☐ Unique biological materials
☒	☐ Antibodies
☒	☐ Eukaryotic cell lines
☒	☐ Palaeontology
☒	☐ Animals and other organisms
☒	☐ Human research participants

Methods

n/a	Involved in the study
☒	☐ ChIP-seq
☒	☐ Flow cytometry
☒	☐ MRI-based neuroimaging
